# Genomic surveillance indicates high site-specific heterogeneity of West Nile virus in mosquitoes in rural regions of Germany across seasons

**DOI:** 10.1016/j.onehlt.2025.101179

**Published:** 2025-08-21

**Authors:** Corinna Patzina-Mehling, Anne Kopp, Leif Rauhöft, Tatiana Șuleșco, Terry C. Jones, Christian Drosten, Felix Gregor Sauer, Renke Lühken, Sandra Junglen

**Affiliations:** a Institute of Virology, Charité - Universitätsmedizin Berlin, Corporate Member of Freie Universität Berlin, Humboldt-Universität zu Berlin and Berlin Institute of Health, Charitéplatz 1, D-10117 Berlin, Germany; b Bernhard Nocht Institute for Tropical Medicine, Bernhard-Nocht-Straße 74, D-20359 Hamburg, Germany; c Centre for Pathogen Evolution, Department of Zoology, University of Cambridge, Cambridge, UK; d German Centre for Infection Research (DZIF), Partner Site Charité, 10117 Berlin, Germany

**Keywords:** West Nile virus, Arbovirus, Mosquito, Germany, Genomic surveillance

## Abstract

The ecology of West Nile virus (WNV) is complex, making it a prime example of the One Health approach. The transmission of WNV to humans depends on multiple factors, such as the presence of amplifying avian hosts and competent mosquito vectors. The abundance of the latter is influenced by land cover, temperature and climate. WNV lineage 2 has been endemic in Europe since around 2004, in Germany since 2018 with recurrent seasonal outbreaks. Endemic areas appear to be relatively stable, such as the Emilia-Romagna region in north-eastern Italy, Macedonia in central Greece and Saxony in eastern Germany. WNV sequences from affected regions are typically monophyletic, indicating regional maintenance. However, there is little knowledge on WNV dispersal and maintenance on a small spatial scale. WNV is maintained in mosquitoes over seasons, but due to a lack of genomic surveillance, it is not known whether this leads to localized persistence of WNV and how endemic virus variants disperse.

In this study, we combined field sampling of adult mosquitoes at a small spatial scale over two seasons with WNV genome analyses, including testing of 15,672 mosquitoes of the genus *Culex* and sequencing of 18 WNV genomes from individual specimens. This study represents a first approach to regional mosquito-based WNV genomic surveillance in rural areas of the WNV endemic area in eastern Germany. Our results show that WNV is present at several sites both within and between two consecutive years with infection rates of up to 0.9 %. WNV sequences fall within the Eastern German clade in phylogenetic analyses but were highly diversified. Surprisingly, genetically different variants were found at same sites, and, in a few cases, genetically more similar variants were found at different sites. These findings suggest that there is extensive dispersal of variants in the region with limited evidence for localized amplification and persistence. We found statistical support for virus dispersal between two sites that are located along a direct local road connection.

Understanding WNV ecology at small spatial scales is crucial to identify patterns of virus maintenance, dispersal and areas with higher WNV abundance rates, and to implement tailored prevention strategies to improve public health.

## Highlights

• Systematic mosquito-based monitoring at small spatial scale reveals high WNV infection rates in a rural endemicregion.
• Phylogenetic analysis revealed enzootic regional maintenance of WNV in eastern Germany in two consecutive years.
• WNV genomic surveillance highlights the dispersal of related virus variants at multiple sites both within and between years.
• Systematic WNV monitoring combined with genomic surveillance is crucial for the understanding of local WNV maintenance.

## 1 Introduction

West Nile virus (WNV) is a mosquito-borne arbovirus of the genus *Flavivirus* that is maintained in a bird-mosquito transmission cycle, typically transmitting the virus between mosquitoes of the *Culex pipiens* complex as main vectors and passerine birds as amplifying hosts [1,2]. The virus can infect a variety of mammalian species including humans and horses, in which it causes disease with viremia levels insufficient for onward transmission to mosquitoes, making these species so-called dead-end hosts [3]. In humans, the majority of infections remain asymptomatic. It is estimated that 20–25 % of infected people develop generalized febrile illness, and less than 1 % progress to neurological manifestation [4,5]. WNV disease transmission depends on complex interactions between the spatial and temporal distribution of hosts and vectors that can amplify and transmit the virus, influenced by temperature, precipitation and the environment.

WNV was first detected in a febrile patient in Uganda in 1937 and has since spread globally, with detections on all continents except Antarctica [6,7]. In Europe, WNV lineage 2 was introduced to Hungary in 2004 [8], subsequently spread to other European countries and is now responsible for the majority of WNV infections in Europe. Since the first detection of WNV lineage 2 in Greece in 2010 [9] and Italy in 2011 [10], autochthonous human cases have occurred annually [[11], [12], [13], [14]]. In 2018, the largest WNV outbreak in Europe with a reported 2083 human cases [15] also entailed the first detection of virus in birds and horses in Germany [16]. The causative WNV strain of lineage 2 was closest to strains from an eastern neighbouring country, Czech Republic, suggesting introduction by migratory birds [16,17]. From the following year onward, the virus was detected in birds, horses, and mosquitoes in eastern Germany [[17], [18], [19], [20]]. Human cases without travel history were seen in the same area from 2019 onward [17,19,[21], [22], [23]]. Phylogenetic analyses revealed close relationship of local WNV sequences, clustering over several subsequent sampling years in a local virus clade that indicates ecological establishment (enzooty) in the region [16,17,19].

The evolution and large-area spread of WNV lineage 2 in Europe has been phylogenetically traced through Italy [24], Greece [25], Germany [17], and the Mediterranean [26]. Agriculture, urbanization and bird habitats were found to be of likely influence on large-area spread [27]. WNV phylogenetic lineages are diverse particularly in southern Europe following repeated introductions by migratory birds [27,28]. Less knowledge is available on local evolution and maintenance of WNV after introduction into new regions.

WNV infection is notifiable for humans and equids in the European Union. Many countries have established WNV surveillance in humans, equines, birds and mosquitoes implementing a One Health approach. In Germany, testing of birds and mosquitoes is mostly covered by research programs and passive veterinary surveillance [[29], [30], [31], [32]]. Human blood and organ donors are subject to seasonally- and locally restricted screening by nucleic acid and antibody testing [33]. This regimen leaves gaps in genomic surveillance that limit our ability to understand local viral dispersal.

Analyses in southern Europe provide evidence for local maintenance and endemic amplification of WNV in the summer [34,35]. WNV is detected in overwintering mosquitoes, confirming enzooty [20,34,36] but it is not well understood whether this facilitates local viral persistence over seasons, or whether the virus uses alternative overwintering strategies such as in vertebrate hosts [37]. Preliminary evidence from Berlin has identified a local virus carrying two signature single nucleotide variants (SNVs; A_7722_G and A_8133_G) that was detected in mosquitoes at one same garden plot colony in two consecutive years, indicating local maintenance in mosquito populations [19]. For a better understanding of local maintenance and dispersal, systematic genomic surveillance in mosquitoes would be crucial. Here we sampled mosquitoes of the genus *Culex* at several sites in eastern Germany, where WNV has been reported annually since 2018 [16,38], over two consecutive years. The aim was to analyse WNV infection rates and distribution based on genomic surveillance.

## 2 Material & methods

### 2.1 Mosquito collection and identification

Mosquitoes were trapped at eight sites in 2021 and seven in 2022, of which six sites were identical in both years. For site eight in 2022, data from two sites situated within two km of each other were merged. All locations were rural in character and consisted of large private properties on the outskirts of villages or outside villages. In 2021, mosquitoes were caught for four nights per month (June–September) using 11–14 BG-Sentinel 2 traps (Biogents, Germany) placed about 25–30 m apart from each other around a control area of approximately one ha. Additionally, artificial resting sites were installed at ground level within the vegetation at the same sites where the BG-Sentinel 2 traps were placed and sampled using an aspirator as described previously [39]. In 2022, mosquitoes were trapped only once for ten nights at the end of July and beginning of August with traps placed approximately 10–40 m apart.

A map of the trapping sites in Germany was created using the QGIS 3.4 software (Open Source Geospatial Foundation; http://qgis.org; 2024) incorporating open-source digital geographic data from the German Federal Office for Cartography and Geodesy (Bundesamt für Kartographie und Geodäsie; https://gdz.bkg.bund.de/; NUTS-Gebiete 1:5000000, Stand 31.12. (NUTS5000 31.12.); accessed 8 May 2024).

### 2.2 Mosquito processing and virus detection by PCR

Mosquitoes of the genus *Culex* were homogenized individually with 500 μL phosphate-buffered saline (PBS) and ceramic beads using the TissueLyser (Qiagen, Germany). For initial screening, pools of ten and super-pools of 100 homogenates were created. RNA was extracted using the MagNA Pure 96 DNA and Viral NA Small Volume Kit (Roche Diagnostics, Germany), and cDNA was synthesized with SuperScript IV reverse transcriptase (Thermo Fisher Scientific, USA) and random hexamer primers (Integrated DNA Technologies, Germany). Two μL of cDNA were then subjected to WNV-specific RT-PCR, including a plasmid-based standard dilution series for viral copy number determination. C_T_-values below 41 of individual mosquitoes were considered as WNV-positive [40].

### 2.3 West Nile virus sequencing and phylogenetic analysis

WNV sequences covering the whole coding region were generated by native and amplicon-based next generation sequencing (NGS). For native NGS, library preparation was performed using the KAPA RNA Hyper Prep Kit (Roche Diagnostics, Germany) and 5 μL RNA of WNV-positive mosquito homogenates with a C_T_-value below 27 (see Table 2). Libraries were quantified using the Qubit dsDNA HS Assay kit (Thermo Fisher Scientific, USA) and the Agilent TapeStation with the HS D1000 Kit (Agilent, Germany). Native NGS was performed on an Illumina NextSeq 2000 platform (Illumina, USA).

For amplicon-based NGS, WNV RNA from the remaining samples was amplified with a multiplex PCR designed using primalscheme [41] for 500 nt amplicons (26 primer pairs, see Supplementary Table S1). For each sample, two PCR mixes were prepared using 2 μL cDNA and Platinum Taq Polymerase (Thermo Fisher Scientific) with 2 mM MgCl_2_, 200 μM dNTPs and 0.062 μM of each primer. Thermal cycling was performed at 95 °C for 3 min, followed by 40 steps of 15 s at 95 °C, 20 s at 60 °C and 30 s at 72 °C. Final elongation was performed for 5 min at 72 °C. Libraries were prepared using the KAPA RNA Hyper Plus Kit (Roche Diagnostics), quantified as described above, and amplicon-based NGS was performed using Illumina MiSeq v3, 600 cycles (Illumina).

WNV consensus sequences were determined using Geneious Prime 9.1.8 (Biomatters, New Zealand) after reads were trimmed using BBDuk (v35.82 by Brian Bushnell) and mapped against the German WNV reference sequence OP810567. The complete coding regions of the novel WNV sequences and publicly available reference sequences from Germany (obtained from the National Center for Biotechnology Information (US) online database) were aligned using MAFFT v7.308 [42]. Following a model test with MEGA v11.0.13, a maximum likelihood (PHYML) tree was inferred using the TN93 substitution model and 1000 bootstrap replicates.

### 2.4 Analysis of association with sampling sites

The new genomes were augmented by six genomes from our previous study in southwestern Berlin [19] as well as two genomes from eastern Berlin [18]. The resulting alignment of 26 mosquito-derived full genomes was augmented by 85 related genomes that defined the last common ancestor clade of the 26 mosquito-derived genomes, as well as an appropriate, closely related phylogenetically and geographically distinct virus (MN481596 – Greece/Bird/2011). With the latter as an outgroup, we used MrBayes under a GTR substitution model with an unconstrained branch length prior, allowing four gamma-distributed substitution rate categories and employing four heated parallel Markov chains [43]. All 84 genomes not pertaining to the initial group of mosquito-derived viruses as well as the outgroup were then removed from the trees. Sample site information was defined as discrete traits for all 27 mosquito-derived genomes. Tree modification and trait reconstruction analysis was done in Mesquite [44]. Trait reconstruction employed and unordered parsimony assumption, allowing up to 50 reconstructions per tree. Summarized state (trait) changes over 600 trees were copied into Excel and z-scores determined for all elements in the trait change matrix, subtracting the mean trait change frequency from the original and 50 different permutations of trait associations from the original trait change frequency, and dividing the result by the standard deviation of the mean (Z = (Obs.-mean)/StdDev).

## 3 Results

**Fig. 1:**
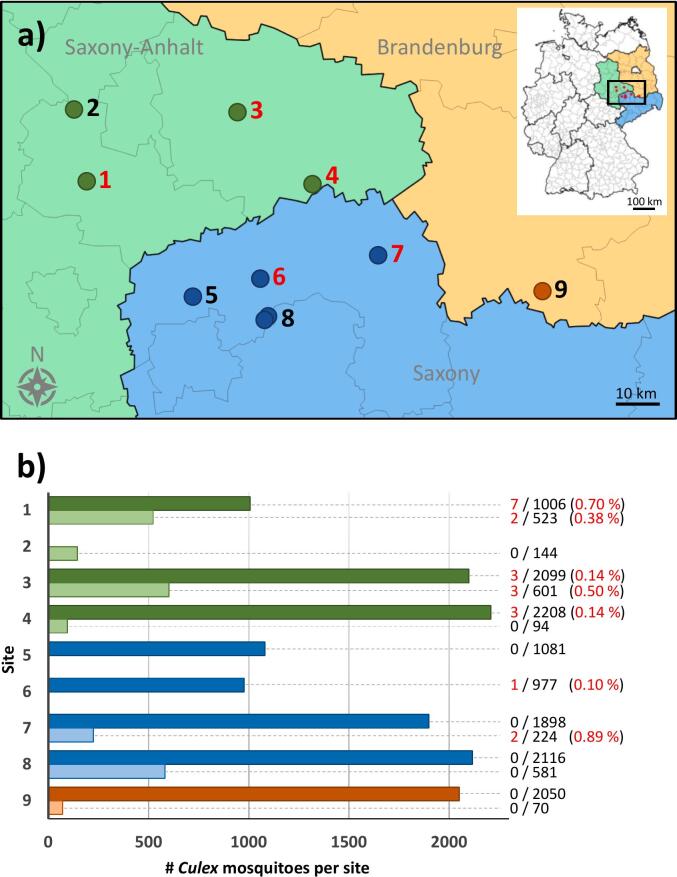
Mosquito collection at individual sites in the WNV endemic region of eastern Germany. (a) Map of the collection sites covering the German federal states of Saxony-Anhalt, Saxony and Brandenburg. Site 8 included two neighbouring sites less than 2 km apart. Sites with WNV-positive mosquitoes are marked in red. (b) The total numbers of mosquitoes caught per site (1–9) in 2021 or 2022 is shown. The number of WNV-positive mosquitoes is highlighted in red, together with the infection rate (IR in %) shown in parentheses. (For interpretation of the references to colour in this figure legend, the reader is referred to the web version of this article.)

The sampling sites were located in three neighbouring German federal states (Saxony-Anhalt, Saxony, and Brandenburg) and covered an area of approximately 40 km by 100 km (in latitude and longitude, respectively), with a maximum distance of ∼100 km, and a minimum distance of ∼10 km between sites. Mosquitoes were collected at six sites from June to September in two consecutive years, with two additional sites in 2021 and one additional site in 2022 (Fig. 1a, Table 1). In total, 15,672 mosquitoes of the genus *Culex* were analysed. The number of mosquitoes varied between sites and years (from 70 to 2208; see Fig. 1b and Table 1). For sites two, five, and six, mosquitoes could only be analysed for one year.

**Table 1:** Site-specific sample statistics.

Site	Latitude	Longitude	Federal state	# mosq 2021	# mosq 2022	# WNV-pos 2021	# WNV-pos 2022	WNV IR 2021 (%)	WNV IR 2022 (%)
1	51.7123	12.067804	Saxony-Anhalt	1006	523	7	2	0.70	0.38
2	51.8597	12.034714	Saxony-Anhalt	n.d.	144	0	0	0	0
3	51.8373	12.566854	Saxony-Anhalt	2099	601	3	3	0.14	0.50
4	51.6866	12.800308	Saxony-Anhalt	2208	94	3	0	0.14	0
5	51.4721	12.395046	Saxony	1081	n.d.	0	0	0	0
6	51.5030	12.613935	Saxony	977	n.d.	1	0	0.10	0
7	51.5358	12.999798	Saxony	1898	224	0	2	0	0.89
8	51.4191	12.618993	Saxony	2116	491	0	0	0	0
8	51.4259	12.636726	Saxony	0	90	0	0	0	0
9	51.4459	13.519128	Brandenburg	2050	70	0	0	0	0

# mosq, analysed number of mosquitoes of the genus *Culex*; # WNV-pos, total number of WNV-positive specimens; IR, infection rate; n.d. = not done.

WNV was detected by RT-PCR in 21 individual mosquitoes, 14 in 2021 and seven in 2022, resulting in infection rates of 0.10 % in 2021 and 0.31 % in 2022. WNV was found in *Culex pipiens* complex (*Culex pipiens* s.s./*Culex torrentium* specimens, *n* = 18), and in three *Culex* spp. mosquitoes (Table 2). WNV was detected at sites one and three in both years, with site-specific WNV infection rates of 0.70 % or 0.14 % in 2021 and 0.38 % or 0.50 % in 2022, respectively (Fig. 1b). At sites four and six, WNV infection rates of 0.14 % and 0.10 % were found in 2021, but WNV was not detected at these sites in 2022, most likely due to low catch numbers at site four (*n* = 94) and no mosquito collection at site six. Of note, WNV was not found at site seven in 2021 despite a high number of mosquitoes analysed (*n* = 1898). Compared to the detection rate of WNV at the other sites, a relatively high infection rate of 0.89 % was detected at site seven in 2022 (2 out of 224), indicative of a new introduction of this virus variant into this site, most likely from site three (see below). WNV was not detected at four sites during our study (sites two, five, eight, and nine).

**Table 2:** Metadata for WNV strains detected in this study.

Strain	Species^⁎^	Gender	Site	Sampling date	C_T_^⁎⁎^	Genome copy numbers^⁎⁎⁎^	Accession #
WNV_M07841	*Culex pipiens* complex	female	1	09-09-2021	19.62	1.86*10^10^	PQ475051
WNV_M07848	*Culex pipiens* complex	female	1	09-09-2021	40.58	3.78*10^2^	partial
WNV_M07912	*Culex* spp.	female	1	09-09-2021	23.25	8.70*10^8^	PQ475052
WNV_M07961	*Culex pipiens* complex	female	1	09-09-2021	21.25	4.72*10^9^	PQ475053
WNV_M08357	*Culex pipiens* Complex	female	1	05-09-2021	31.46	8.40*10^5^	PQ475054
WNV_M11788	*Culex pipiens* complex	female	1	11-08-2021	23.80	1.87*10^8^	PQ475061
WNV_M11803	*Culex pipiens* complex	female	1	11-08-2021	34.09	2.70*10^5^	PQ475062
WNV_M10302	*Culex pipiens* complex	female	1	31-07-2022	22.08	5.58*10^8^	PQ475060
WNV_M13782	*Culex pipiens* complex	female	1	04-08-2022	22.97	3.18*10^8^	PQ475065
WNV_M09233	*Culex* spp.	female	3	12-07-2021	23.27	8.50*10^8^	PQ475055
WNV_M09577	*Culex* spp.	female	3	13-08-2021	29.34	5.04*10^6^	PQ475057
WNV_M09897	*Culex pipiens* complex	female	3	13-08-2021	30.95	1.99*10^6^	PQ475058
WNV_M13565	*Culex pipiens* complex	female	3	04-08-2022	38.68	1.47*10^4^	partial
WNV_M13615	*Culex pipiens* complex	female	3	04-08-2022	23.60	2.12*10^8^	PQ475063
WNV_M13617	*Culex pipiens* complex	female	3	04-08-2022	26.12	4.30*10^7^	PQ475064
WNV_M04183	*Culex pipiens* complex	female	4	05-09-2021	24.61	2.74*10^8^	PQ475049
WNV_M06800	*Culex pipiens* complex	female	4	13-08-2021	30.37	2.12*10^6^	PQ475050
WNV_M10186	*Culex pipiens* complex	female	4	10-08-2021	19.80	2.38*10^9^	PQ475059
WNV_M09555	*Culex pipiens* complex	female	6	06-09-2021	25.51	1.29*10^8^	PQ475056
WNV_M15698	*Culex pipiens* complex	female	7	05-08-2022	21.87	6.38*10^8^	PQ475066
WNV_M15703	*Culex pipiens* complex	female	7	05-08-2022	39.91	6.72*10^3^	partial

*Culex pipiens complex encompasses the species *Culex pipiens* s.s. and *Culex torrentium*; **RT-PCR threshold cycle; ***RNA copies per 500 μL of mosquito homogenate.

**Fig. 2:**
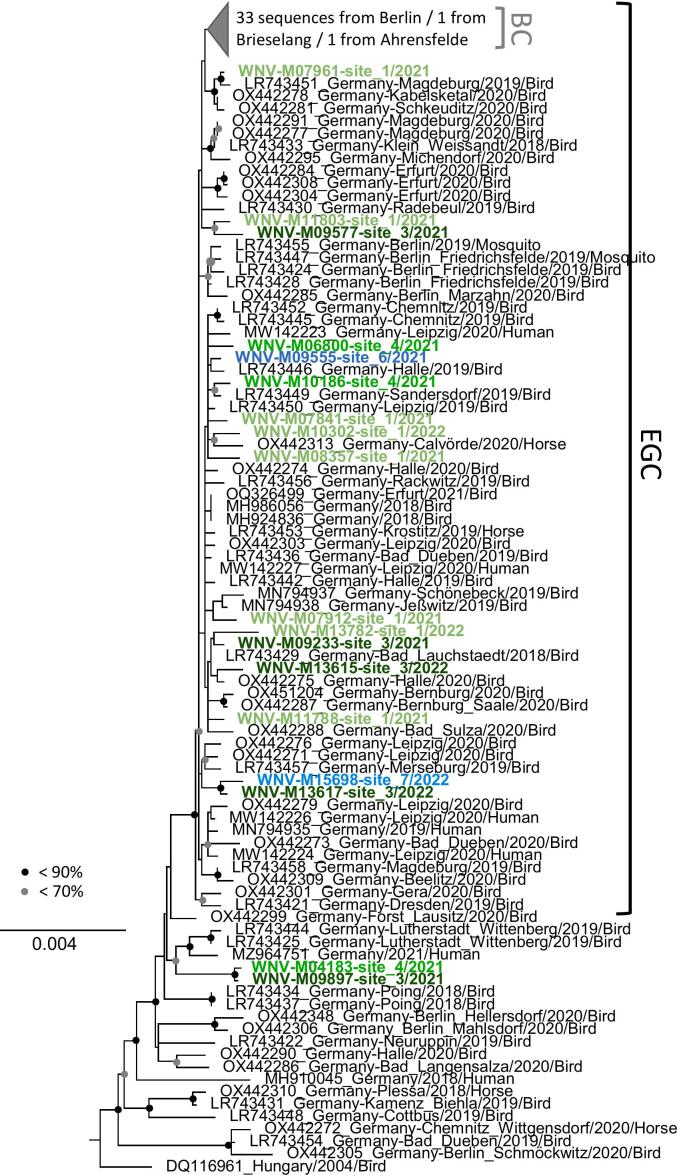
Phylogenetic analysis of the 18 WNV sequences from this study (highlighted in green (Saxony-Anhalt) and blue (Saxony), with different shading per site. Eleven sequences shared two nucleotide variations (C3615T and C4041T) with a subset of WNV sequences from the eastern German clade (EGC). The Berlin subclade is shown in dark grey. The maximum likelihood (PHYML) tree was inferred from a MAFFT alignment using the TN93 substitution model and 1000 bootstrap replicates (bootstrap values above 50 are shown). (For interpretation of the references to colour in this figure legend, the reader is referred to the web version of this article.)

In total, 18 coding-complete WNV genomes were successfully generated from individual mosquitoes and phylogenetically analysed (Table 2; GenBank accession numbers PQ475049-PQ475066). The sequences showed high site-specific heterogeneity with up to 51 SNVs per site. The eight WNV genomes obtained from site one showed 13–27 SNVs, the five from site three 14–45 SNVs, the three from site four 15–45 SNVs. All sequences belonged to WNV lineage 2 and grouped with WNV sequences from birds, horses, humans, and mosquitoes from Germany (Fig. 2). Attempts to resolve the topological uncertainty within the German clade using different substitution models were not successful. Similar sequences were not detected at the same site and not within and across seasons but were found at different sites. For example, similar sequences were detected at sites one and three (WNV-M11803-site 1/2021 and WNV-M09577-site 3/2021), sites three and four (WNV-M09897-site 3/2021 and WNV-M04183-site 4/2021), as well as sites three and seven (WNV-M13617-site 3/2022 and WNV-M15698-site 7/2022) (Fig. 2).

To determine sampling site association, we looked for signs of incoherence between site-specific segregation and tree topology for mosquito-derived full genomes based on a parameter-rich Bayesian reconstruction of phylogeny. Parsimony reconstruction of trait changes yielded signals indicating statistically significant changes of trait associations for a change from site three to site seven (z-score 1.99, Supplementary Table S2). This matches the observation of new occurrence of virus at site seven in the second sampling year (the formal analysis was agnostic of time).

## 4 Discussion

This study represents a first attempt at local mosquito-based genomic surveillance for WNV in rural areas of an endemic region in the eastern part of Germany, with the aim of improving our understanding of the local dispersal of endemic WNV variants at small geographical scales.

Our findings indicate WNV was present at multiple sites in two consecutive years, with total WNV IRs of 0.10 and 0.31 % for 2021 or 2022, respectively. These results are in line with other studies. For example, WNV minimal infection rates in samples from a closeby urban area (metropolitan Berlin) ranged from 0.17 to 0.35 % [18,19]. In other endemic regions in southern Europe, such as Emilia-Romagna in Italy and Central Macedonia in Greece, minimal infection rates in mosquitoes were only slightly higher, ranging from 0.19 to almost 2 % [[45], [46], [47]], suggesting a comparable endemic situation for WNV in these different European regions. Among our study sites we found site-specific infection rates to vary from 0.10 to 0.89 %, with some sites remaining negative throughout our sampling and three sites positive in one of two seasons only, suggesting uneven dispersal.

Phylogenetic analysis revealed the close relationship of the WNV strains from this study with other sequences from eastern Germany, 16 of those clustering within the previously defined eastern German clade [16,17], and two falling outside this clade but also grouping with sequences previously described from the region [48,49]. This implies perennial enzootic maintainance and no obvious introduction of novel lineages from outside the regions. This is further supported by eleven sequences sharing two marker mutations within the eastern German clade, indicative of ongoing regional evolution of the predominant WNV variants and their maintenance within bird and mosquito populations in the region. Similar establishments of regional hotspots with genomic clustering and regional maintenance of predominant WNV variants have been described for Emilia-Romagna in Italy [34], as well as Central Macedonia in Greece [35].

At the same time, WNV sequences from the present study exhibit considerable genetic diversity. We detected genetically distinct variants at the same locations, and in some cases, more genetically similar variants at different sites. This suggests intermittent dispersal within the region that is superimposed on local lineage maintenance and local microevolution seen before in the region of study [19,20]. We find evidence of a component of local-area movement that seemed to correlate with road connections between sampling sites, rather than geographic proximity (Fig. 1, Fig. 2). Sites three and seven are connected via L182, the shortest road connection between the cities of Riesa and Wittenberg (ca. 88.5 km).

Our study has limitations in terms of sample size and evenness. Negative findings cannot prove viral absence in a given site. Total number of *Culex* mosquitoes captured per site ranged as wide as 70 to 2208 specimens. The very dry 2022 summer likely caused a decrease in the number of mosquitoes collected. Low mosquito numbers from some sites, as well as an overall low catch in the arid summer 2022 [50] may affect the reliability of a direct comparison. Also, phylogeographic analysis was agnostic of branch lengths and thus did not attribute differential weights to outlying lineages. We put emphasis on topology and its uncertainty as the overall phylogenetic tree already suggested regional stability.

So far, little is known about bird species acting as amplifying hosts and reservoir of the virus in central Europe. Surveillance typically focuses on captive (sentinel) birds or cadavers submitted for veterinary investigation [[30], [31], [32]]. This neglects potential natural reservoir species that typically display high viremia and mild symptoms such as in the case of American robins in North America [3,51]. Our observations are compatible with the concept of lineage dispersal by movement of infected birds, which is likely higher in the here-examined rural areas than in the metropolitan area of Berlin. Urban-adapted bird populations are more residential [[52], [53], [54], [55]], matching the pattern of local virus maintenance in urban Berlin [19]. An alternative hypothesis for regional virus dispersal is human influence. In the present study, those two sites with statistical support for virus dispersal are located along a direct local road connection. Even though viral maintenance over perennial cycles in *Culex* is not deemed to occur in the form of eggs but rather in hibernating mosquitoes, dispersal of living mosquitos with short-distance road traffic is an option to be investigated in future studies.

## 5 Conclusion

Overall, this study implemented a WNV genomic surveillance approach at small spatial scale that highlights the widespread dispersal of genetically related WNV variants in a rural endemic hotspot region in eastern Germany in the consecutive years 2021 and 2022, including high rates of WNV IRs in local mosquitoes. Of note, WNV variants from this study showed no evidence of local persistence at small spatial scales, but rather of frequent exchange between sites. At the investigated sites, WNV small-scale ecology and maintenance seems to differ from an urban site in Berlin. Systematic WNV monitoring combined with genomic surveillance would be crucial for the understanding of the establishment and maintenance of local hotspots and thus the identification of areas of increased risk for human exposure and infection.

## Author contribution

CPM, FGS, RL and SJ designed and conceptualized the study. LR, TS, FGS and RL conducted the fieldwork including mosquito identification. CPM, AK and SJ contributed to data collection, laboratory analysis, interpretation and visualization. TCJ and CD conducted data analysis, interpretation and visualization. CPM and SJ were involved in the writing of the original draft. All authors contributed to the review and editing process, and approved the final version of the manuscript.

## Declaration of competing interest

The authors declare no conflict of interest.

## Data Availability

Sequence information is publicly available at GenBank under the accession numbers PQ475049-PQ475066.

## Appendix A Supplementary data

Supplementary material Image 1
