# Supplementary material for: Genomic surveillance indicates high site-specific heterogeneity of West Nile virus in mosquitoes in rural regions of Germany across seasons
Source: One Health. 2025 Aug 21;21:101179. doi: 10.1016/j.onehlt.2025.101179 (PMC12765102; doi:10.1016/j.onehlt.2025.101179)
Supplement: Supplementary file 1 — Supplementary material [file mmc1.docx]

**Supplementary Material**

**Table S1:** Primers for whole genome amplification pools 1 or 2 of the coding region of WNV by multiplex PCR.

| **NAME** | **POOL** | **SEQUENCE** |
| --- | --- | --- |
| 01F_DE_WNV_lin2 | 1 | AACAATTAACACAGTGCGAGCTG |
| 01R_DE_WNV_lin2 | 1 | CACAAAGGTATCCCACGTCCAT |
| 02F_DE_WNV_lin2 | 2 | TGATGACAGTCAATGCAACCGA |
| 02R_DE_WNV_lin2 | 2 | TCCCTCCAGGAAGTCTCTGTTAC |
| 03F_DE_WNV_lin2 | 1 | AATGCAACGCGTCGTGTTTG |
| 03R_DE_WNV_lin2 | 1 | ATGAGATTCAACAGTCGTCGGG |
| 04F_DE_WNV_lin2 | 2 | GCCTGTACAACCAAAGCAACTG |
| 04R_DE_WNV_lin2 | 2 | CTTCACCCTACACTTCAGATGTCC |
| 05F_DE_WNV_lin2 | 1 | CAATCTGTTGTGGCTCTAGGGTC |
| 05R_DE_WNV_lin2 | 1 | TCGTTGAGCTCCTCTGAGTGTA |
| 06F_DE_WNV_lin2 | 2 | TGGGAAGAGGAGAACAGCAGAT |
| 06R_DE_WNV_lin2 | 2 | CTCCTTCTGCATGTGCTTTCTGA |
| 07F_DE_WNV_lin2 | 1 | TGGAAGCCTGGATGGATCGTTA |
| 07R_DE_WNV_lin2 | 1 | GCGGTTCCTATGATCTTCGAGTC |
| 08F_DE_WNV_lin2 | 2 | GGATTTGGATTGACAAGCACTCG |
| 08R_DE_WNV_lin2 | 2 | CATTCTCAGTCTGGAAGCGCA |
| 09F_DE_WNV_lin2 | 1 | TTGCCCAGGAACAACAGTAACT |
| 09R_DE_WNV_lin2 | 1 | AAGGAAGCCACCAGAAAGACTG |
| 10F_DE_WNV_lin2 | 2 | TGCGATATGTCATTCTCGTCGG |
| 10R_DE_WNV_lin2 | 2 | CCCAGCTGCAAGTATCATTGGA |
| 11F_DE_WNV_lin2 | 1 | GTTGGAGTTGGAAGCCTCATCA |
| 11R_DE_WNV_lin2 | 1 | CCAATGACCGAGGGAAGAATCG |
| 12F_DE_WNV_lin2 | 2 | AATTGATAAATGACCCCGGGGC |
| 12R_DE_WNV_lin2 | 2 | ACTGCCCCAATTTCTCCTTCTG |
| 13F_DE_WNV_lin2 | 1 | TGGAATGGACATGATGAGGTC |
| 13R_DE_WNV_lin2 | 1 | ACGATCTCATTTCCACTGTGCTC |
| 14F_DE_WNV_lin2 | 2 | TGAGAGGACTTCCCATCCGGTA |
| 14R_DE_WNV_lin2 | 2 | CATCGTTCTTGCACTTGGGGTA |
| 15F_DE_WNV_lin2 | 1 | AATGAGATTGCCCTCTGTCTGC |
| 15R_DE_WNV_lin2 | 1 | AGTTCTTCCGTTCTTCCCCTCT |
| 16F_DE_WNV_lin2 | 2 | AACATGCCGAATGGTCTGGTG |
| 16R_DE_WNV_lin2 | 2 | TCAAGAGCCATCCTGTGAGC |
| 17F_DE_WNV_lin2 | 1 | CCTGAGCACTTCATGGGGAAAA |
| 17R_DE_WNV_lin2 | 1 | CCCAGGGTCGTCTCTCTAGTTT |
| 18F_DE_WNV_lin2 | 2 | CAATGAAATGGGTTGGCTGGAC |
| 18R_DE_WNV_lin2 | 2 | AGTTGTTCGCTCAAGTTCAGGT |
| 19F_DE_WNV_lin2 | 1 | ATGCTTACATGGTACCAGGCTG |
| 19R_DE_WNV_lin2 | 1 | GTCGACTTCAGTGATGGCTTCT |
| 20F_DE_WNV_lin2 | 2 | AGGGGAAGTTTGGAAGGAGAGA |
| 20R_DE_WNV_lin2 | 2 | CTTCCACCATCTCCAAGACACG |
| 21F_DE_WNV_lin2 | 1 | CACACTGCTCTGTGACATTGGA |
| 21R_DE_WNV_lin2 | 1 | TGGTAGTTCCAGGTCCTGTAGG |
| 22F_DE_WNV_lin2 | 2 | CAAGAACCGAATTGAGAGGCTGA |
| 22R_DE_WNV_lin2 | 2 | TCCTCATCCACCATCTCCCAAA |
| 23F_DE_WNV_lin2 | 1 | GTCAACAGTAATGCCGCCCT |
| 23R_DE_WNV_lin2 | 1 | GACTTTGTGTCGGTATGTGAGCT |
| 24F_DE_WNV_lin2 | 2 | ATCACCAAAGCTGACCTCGAGA |
| 24R_DE_WNV_lin2 | 2 | TTGAACAGAATGGGACCTGCTG |
| 25F_DE_WNV_lin2 | 1 | CTAAATGCCATGTCAAAGGTCCG |
| 25R_DE_WNV_lin2 | 1 | TGGAACATCACTCCACCTCTCA |
| 26F_DE_WNV_lin2 | 2 | ATGACGACAGAAGACATGCTCG |
| 26R_DE_WNV_lin2 | 2 | GATCACCTCGCAACTTTGGTCA |

**Table S2:** Trait change statistics.
